# Longitudinal circulating tumour DNA dynamics predict failure patterns and efficacy of consolidation immunotherapy after chemoradiotherapy in locally advanced non‐small‐cell lung cancer

**DOI:** 10.1002/ctm2.1619

**Published:** 2024-03-07

**Authors:** Yu Wang, Tao Zhang, Yin Yang, Jianyang Wang, Canjun Li, Xin Xu, Yuqi Wu, Ying Jiang, Jinghao Duan, Luhua Wang, Nan Bi

**Affiliations:** ^1^ Department of Radiation Oncology, National Cancer Center/National Clinical Research Center for Cancer/Cancer Hospital Chinese Academy of Medical Sciences and Peking Union Medical College Beijing China; ^2^ Department of Radiation Oncology, National Cancer Center/National Clinical Research Center for Cancer/Cancer Hospital & Shenzhen Hospital Chinese Academy of Medical Sciences and Peking Union Medical College Shenzhen China; ^3^ State Key Laboratory of Molecular Oncology, National Cancer Center/National Clinical Research Center for Cancer/Cancer Hospital Chinese Academy of Medical Sciences and Peking Union Medical College Beijing China

Dear Editor,

Responses to consolidation immune checkpoint inhibitor (ICI) in locally advanced non‐small‐cell lung cancer (LA‐NSCLC) are heterogeneous, and current decision‐making procedures have little accuracy.^1^ This prospective cohort study provided the first evidence for dynamic circulating tumour DNA (ctDNA) predicting failure patterns in LA‐NSCLC patients receiving chemoradiotherapy (CRT), allowing the early identification of the potentially curable population with radical CRT and different therapeutic benefits from consolidation ICI.

Exploring effective biomarkers to guide personalized consolidation immunotherapy, avoid overtreatment, and reduce the potential risk of immune‐related toxicities is of clinical importance.^1^, ^2^, ^3^ In this prospective multicenter trial (NCT04014465), 105 patients with unresectable LA‐NSCLC were assigned to CRT or CRT plus consolidation ICI cohorts, with balanced baseline characteristics (Figure 1A and Table S1). As expected, patients undergoing consolidation ICI had significantly improved overall and progression‐free survival (PFS) (Figure 1B,C). All patients have collected blood samples at baseline, on‐CRT (radiotherapy reached 40 Gy/4 weeks), post‐CRT (1 month after CRT), and progressive timepoints, subjected to 486‐gene next‐generation sequencing to analyze longitudinal ctDNA. Detailed information about ctDNA assay techniques and study procedures is attached as the Supporting Information. No significant difference in ctDNA abundance across cohorts at any time point (Figure 1D). Notably, quantitative ctDNA could reflect tumour burden,^4^ since ctDNA levels significantly decreased with effective CRT but increased at disease progression, and baseline ctDNA positively correlated with the clinical stage (Figure 1E–G).

**FIGURE 1:**
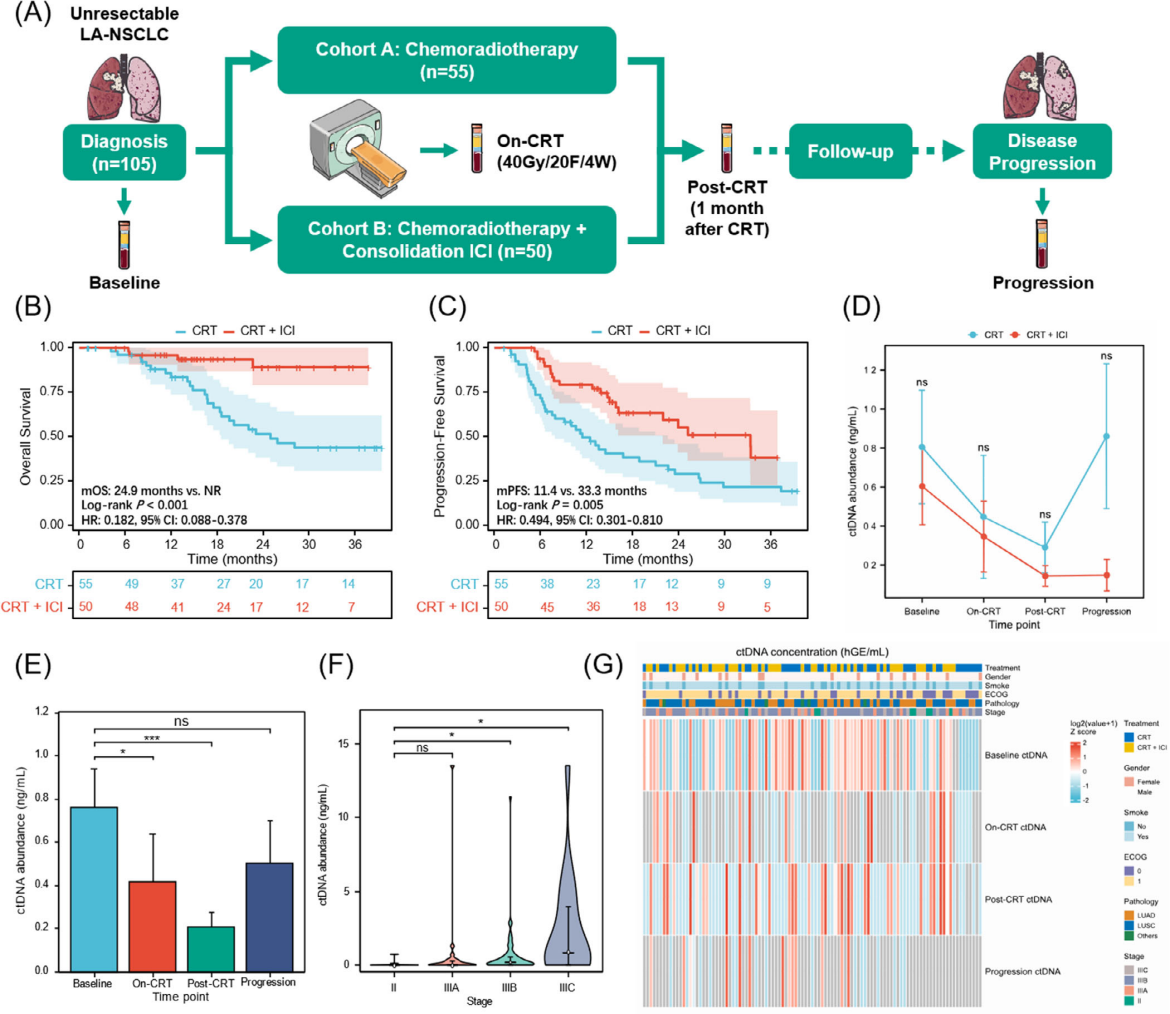
Study design and circulating tumour DNA (ctDNA) detection at longitudinal time points. (A) Study schematic of patient enrolment, treatment regimens, and longitudinal ctDNA monitoring for two cohorts. (B) Kaplan‐Meier analysis of overall survival (OS) stratified by patients with chemoradiotherapy (CRT) (*n* = 55) versus with CRT + consolidation immune checkpoint inhibitor (ICI) (*n* = 50). (C) progression‐free survival (PFS) stratified by patients with CRT (*n* = 55) versus with CRT + consolidation ICI (*n* = 50). (D) Line charts indicating no significant difference in ctDNA abundance between the CRT and CRT + ICI cohorts at neither baseline, on‐CRT, post‐CRT, nor first‐time progression time points. Mean ± SD (standard deviation) is shown. (E) Bar histograms describing ctDNA abundance at baseline, on‐CRT, post‐CRT, and at progression. Mean ± SE (standard error) is shown, and error bars represent SE. ctDNA abundance significantly decreased post‐CRT but increased at the time of disease progression. Significance was calculated using the Mann‐Whitney test. (F) Violin plots demonstrating baseline ctDNA abundance positively correlated with disease stage. Median ± IQR (interquartile range) is shown. (G) Heatmap of ctDNA concentrations for patients in both cohorts at baseline, on‐CRT, post‐CRT, and progression time points. The top heat maps depict key patient characteristics. CI, confidence interval; mOS, median overall survival; mPFS, median progression‐free survival; NR, not reached; HR, hazard ratio; LUAD, lung adenocarcinoma; LUSC, lung squamous carcinoma. ns, non‐significant difference; *p < .05; ***p < .001.

We further explored the prognostic value of ctDNA at longitudinal landmark timepoints. Post‐CRT detectable ctDNA, rather than baseline or on‐CRT ctDNA, was associated with significantly worse survival (Figure 2A–C). In patients with detectable ctDNA post‐CRT, 75% of non‐progression patients received consolidation ICI, while 63.3% of non‐progression patients used ICI in the undetectable population (Figure 2D,E). Survival analyses were confirmed in patients with detectable ctDNA post‐CRT, those receiving consolidation ICI had significantly longer PFS than those without ICI, yet no significant difference in patients with ctDNA clearance (Figure 2F,G). According to post‐CRT ctDNA minus baseline ctDNA levels, the dynamic change pattern of longitudinal ctDNA included decreased (*n* = 54), stably undetectable (*n* = 30), and increased (*n* = 19; Figure 2H). Strikingly, the majority of patients with increased ctDNA (73.7%) developed disease progression (Figure 2I).

**FIGURE 2:**
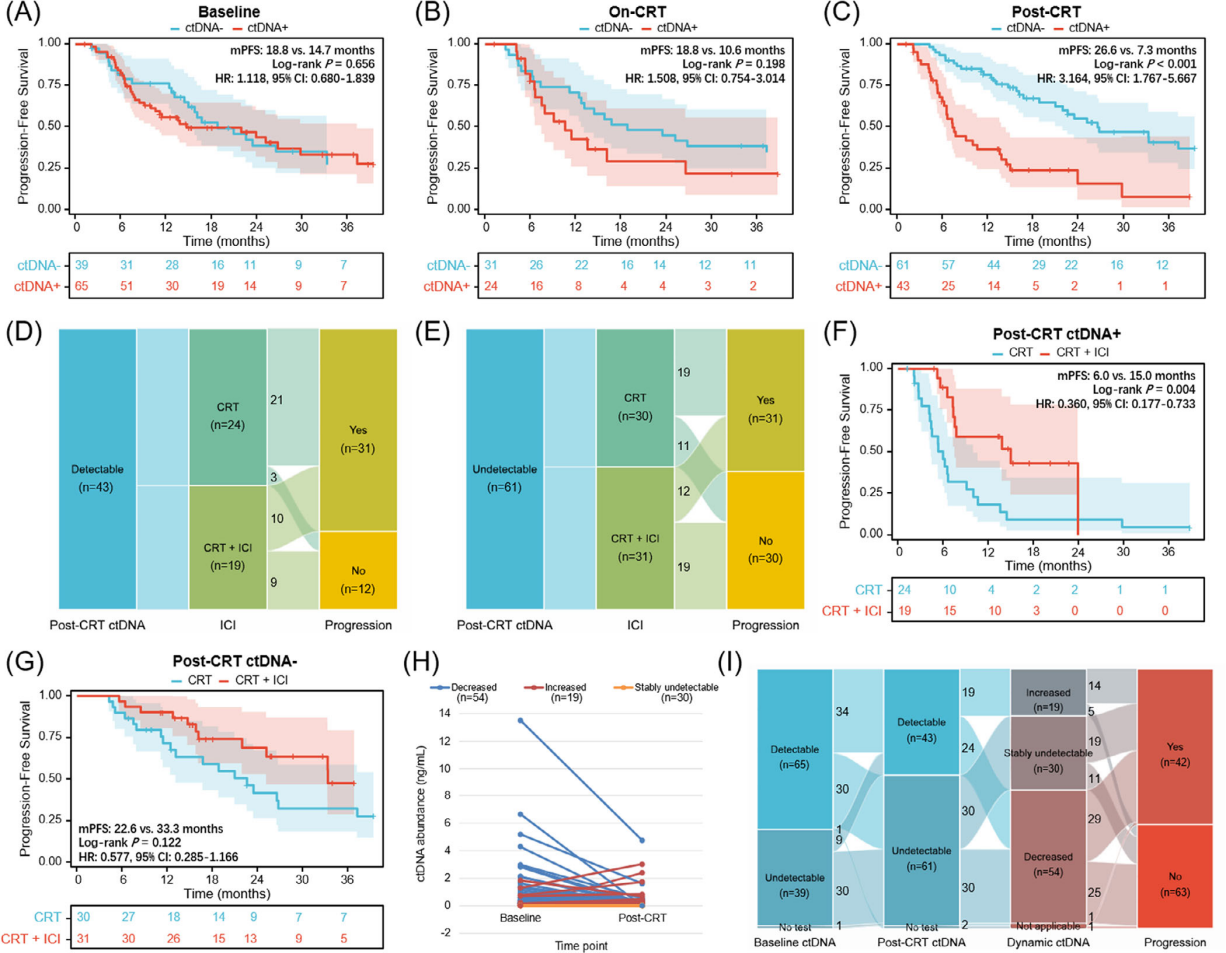
Prognostic and predictive effects of circulating tumour DNA (ctDNA) at the post‐chemoradiotherapy (post‐CRT) timepoint. (A) Progression‐free survival (PFS) stratified by patients with undetectable (*n* = 39) versus detectable (n = 65) baseline ctDNA. (B) PFS stratified by patients with undetectable (*n* = 31) versus detectable (n = 24) on‐CRT ctDNA. (C) PFS stratified by undetectable (*n* = 61) versus detectable (*n* = 43) post‐CRT ctDNA. (D) Sankey plot depicting the proportion of consolidation immune checkpoint inhibitor (ICI) and disease progression status for patients with detectable ctDNA post‐CRT. (E) Sankey plot depicting the proportion of consolidation ICI and disease progression for patients with undetectable ctDNA post‐CRT. (F) PFS stratified by CRT (*n* = 24) versus CRT plus consolidation ICI (*n* = 19) in patients with detectable post‐CRT ctDNA. (G) PFS stratified by CRT (*n* = 30) versus CRT and ICI (*n* = 31) in patients with undetectable post‐CRT ctDNA. (H) Paired scatter plots indicating three dynamic ctDNA patterns (decreased, increased, and stably undetectable) based on post‐CRT minus baseline ctDNA levels. (I) Sankey plot illustrating the proportion of dynamic ctDNA patterns and disease status.

Next, we investigated the predictive effect of longitudinal ctDNA on first failure patterns, which were categorized as local‐regional, distant, or both, as presented in Figure 3A. The primary failure pattern for patients with decreased ctDNA in the CRT cohort was distant metastasis (51.9%), and consolidation ICI significantly reduced the incidence of distant metastasis (18.5%; *p* = .019). However, in the stably undetectable ctDNA group, the incidence of local‐regional failure (21.4% vs. 37.5%) and distant metastasis (28.6% vs. 31.3%) was both similar between patients with and without consolidation ICI (*p* = .592). In the increased ctDNA group, simultaneously local‐regional and distant failure was the most predominant failure pattern (36.4%) for patients with CRT, and consolidation ICI brought a tendency (*p* = .583) of a reduced proportion of local‐regional (18.2% vs. 12.5%), distant (27.3% vs. 25.0%), and simultaneously local‐regional and distant (36.4% vs. 12.5%) failure.

**FIGURE 3:**
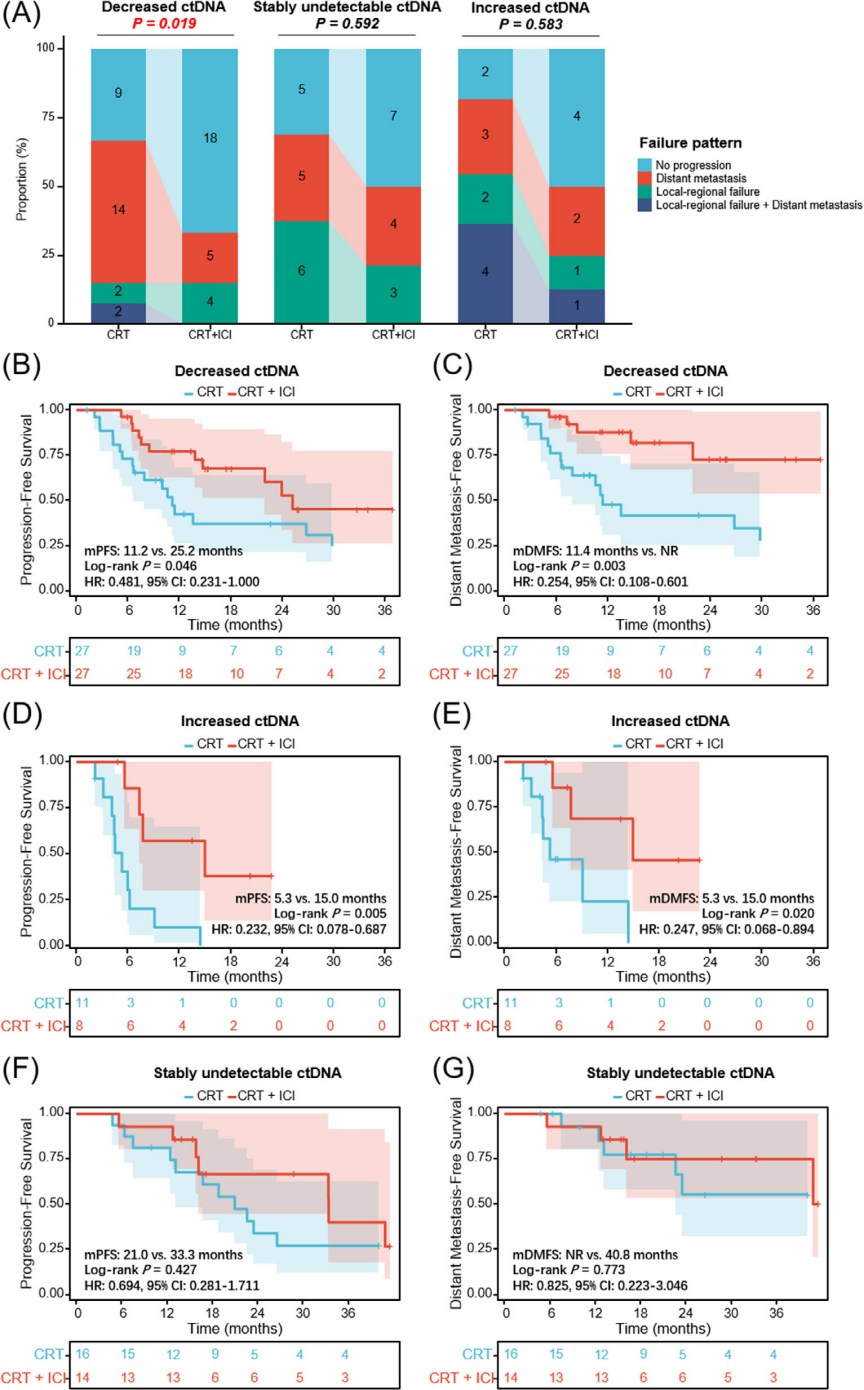
Dynamic circulating tumour DNA (ctDNA) identifies different treatment failure patterns and predicts benefits from consolidation immune checkpoint inhibitor (ICI). (A) The proportion of different first failure patterns for patients with decreased, stably undetectable, and increased ctDNA dynamics in the chemoradiotherapy (CRT) versus CRT and consolidation ICI cohorts. Progression‐free survival (PFS) (B) and DMFS (C) stratified by CRT alone (*n* = 27) versus CRT + consolidation ICI (*n* = 27) cohorts in patients with decreased ctDNA after CRT. PFS (D) and DMFS (E) stratified by CRT alone (*n* = 11) versus CRT + consolidation ICI (*n* = 8) in patients with increased ctDNA. PFS (F) and DMFS (G) stratified by CRT alone (*n* = 16) versus CRT + consolidation ICI (*n* = 14) cohorts in patients with stably undetectable ctDNA. mDMFS, median distant metastasis‐free survival.

For patients with decreased ctDNA post‐CRT, although consolidation ICI brought significant PFS benefit (*p* = .046; Figure 3B), a more obvious distant metastasis‐free survival (DMFS) benefit was observed (*p* = .003; Figure 3C). In the increased ctDNA population (Figure 3D,E), patients with consolidation ICI had significantly improved PFS (*p* = .005) and DMFS (*p* = .020). In contrast, in the stably undetectable ctDNA group (Figure 3F,G), no significant difference in PFS (*p* = .427) or DMFS (*p* = .773) between patients with and without ICI. These data suggest that dynamic ctDNA could identify different failure patterns and therapeutic responses to ICI: 1) Decreased ctDNA dynamics predict a higher risk of distant metastasis but more DMFS benefit from ICI, presumably because effective radiotherapy could cause localized tumour cell death and accordingly decreased release of tumour‐derived products into the peripheral blood, whereas it is difficult for local radiotherapy to completely eradicate disseminated ctDNA pre‐existed in the circulation.^5^ Consolidation ICI as a potent systemic treatment would effectively reduce the risk of overt metastases and micrometastases, thereby bringing DMFS benefit to this patient subset; 2) Increased ctDNA, reflecting resistance to definitive CRT, is associated with a higher risk of distant and local‐regional failure, and subsequent ICI improves DMFS as well as PFS. In the clinical setting, for patients with solitary lesions of suspected metastasis,^6^ dynamic ctDNA testings may contribute to differential diagnosis between primary and metastatic diseases, as increased ctDNA indicates the hematogenous spread of tumour cells in the peripheral circulation^7^ 3) Stably undetectable ctDNA predicts excellent outcomes irrespective of consolidation ICI, suggesting the potentially curable population with CRT.^2^

Cox regression analysis demonstrated consolidation ICI, post‐CRT ctDNA, and dynamic ctDNA changes were significant variables in predicting PFS (Table S2 and Figure S1). Lastly, we compared the prediction effects and clinical decision‐making benefits of single‐time ctDNA detection with longitudinal ctDNA dynamics. Time‐dependent receiver operating characteristic curves indicated that post‐CRT ctDNA was the optimal predictor of PFS (Figure 4A,B). In terms of dynamic ctDNA assessments, as shown in Figure 4C,D, patients with increased ctDNA had the significantly worst PFS and DMFS, and patients with stably undetectable ctDNA had the longest survival. Furthermore, decision curve analysis suggested the combined model integrating qualitative post‐CRT ctDNA detection with quantitative ctDNA dynamics had superior usefulness in predicting PFS and DMFS, compared to individual features (Figure 4E,F). Based on this combinatorial model, patients with decreased ctDNA were further divided into two groups, and patients with post‐CRT detectable/decreased ctDNA had significantly worse PFS and DMFS than those with undetectable/decreased ctDNA (Figure 4G‐H), in both CRT and CRT + consolidation ICI cohorts (Figure S2).

**FIGURE 4:**
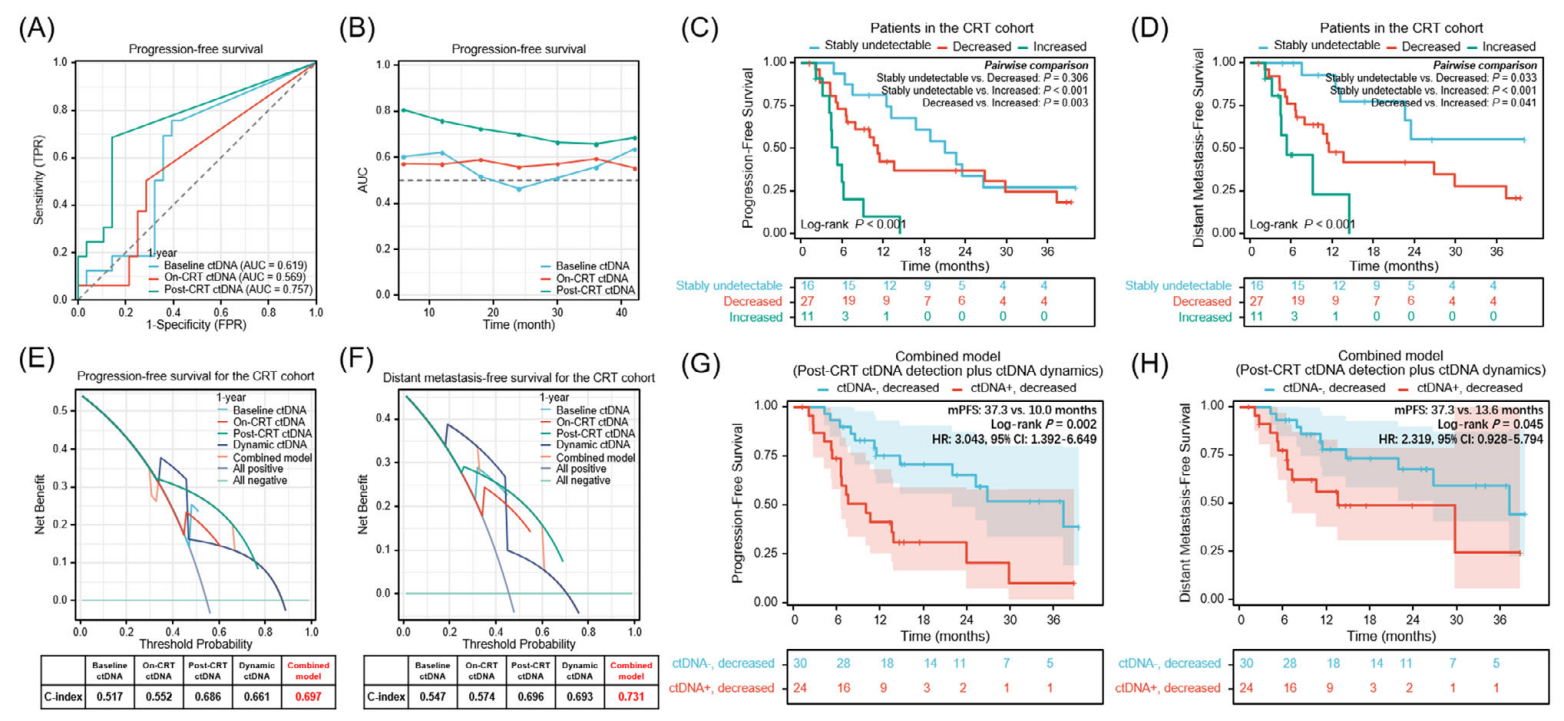
Predictive value of combining post‐chemoradiotherapy (post‐CRT) residual circulating tumour DNA (ctDNA) detection with longitudinal ctDNA dynamics. (A) Time‐dependent receiver operating characteristic curves illustrating the prediction effect of baseline, on‐CRT, and post‐CRT ctDNA levels on progression‐free survival (PFS). (B) Line charts showing AUCs of baseline, on‐CRT, and post‐CRT ctDNA over time. PFS (C) and DMFS (D) stratified by patients with decreased (*n* = 27), stably undetectable (*n* = 16), and increased ctDNA (*n* = 11) in the CRT cohort. Depiction of C‐index, calculated by decision curve analyses, comparing the clinical benefit of baseline, on‐CRT, post‐CRT, dynamic ctDNA, and the combined model (residual ctDNA detection plus dynamic ctDNA) for predicting PFS (E) and DMFS (F) in the CRT cohort. PFS (G) and DMFS (H) stratified by patients with detectable/decreased ctDNA (*n* = 24) versus patients with undetectable/decreased ctDNA (*n* = 30) after CRT. TPR, true positive rate; FPR, false positive rate; AUC, area under the curve; C‐index, concordance index.

In conclusion, we determined that the multiparameter assay integrating qualitative with quantitative ctDNA metrics could improve patient risk stratification, consistent with previous findings.^8^, ^9^, ^10^ Moreover, we innovatively discovered dynamic ctDNA could predict failure patterns, potential curability with CRT, and different responses to consolidation ICI, thereby serving as a robust prognostic and predictive model with improved usefulness to facilitate ctDNA‐guided treatment personalization. Our findings warrant independent validation in a larger‐scale clinical cohort. The ongoing randomized, phase II trial (InTRist, NCT05888402) will further validate the predictive value of longitudinal ctDNA dynamics and provide high‐quality evidence for translational application of ctDNA in LA‐NSCLC.

## AUTHOR CONTRIBUTIONS

Yu Wang and Tao Zhang performed data analysis and interpretation, investigation, as well as manuscript drafting. Yin Yang, Jianyang Wang, Canjun Li, Xin Xu, Yuqi Wu, Ying Jiang and Jinghao Duan assisted with data acquisition and curation, project administration, and independent validation. Luhua Wang and Nan Bi supervised this study, provided resources, acquired funding, and critically revised the manuscript. All authors contributed to the study conceptualization and approved the final version.

## CONFLICT OF INTEREST STATEMENT

The authors declare no conflict of interest.

## FUNDING INFORMATION

This work was founded by the National Natural Sciences Foundation Key Program (No. 82173348), CAMS Innovation Fund for Medical Sciences (No. 2021‐1‐I2M‐1‐012), and the Special Research Fund for Central Universities, Peking Union Medical College (No. 3332023133).

## ETHICS STATEMENT

This study was approved by the institutional review board of the Chinese Academy of Medical Sciences (No. 19/098‐1883). All patients provided written informed consent.

## Supporting information

Supporting Information

Supporting Information

Supporting Information

Supporting Information

Supporting Information
