# Supplementary material for: Longitudinal circulating tumour DNA dynamics predict failure patterns and efficacy of consolidation immunotherapy after chemoradiotherapy in locally advanced non‐small‐cell lung cancer
Source: Clin Transl Med. 2024 Mar 7;14(3):e1619. doi: 10.1002/ctm2.1619 (PMC10918705; doi:10.1002/ctm2.1619)
Supplement: Supplementary file 1 — Supporting Information [file CTM2-14-e1619-s001.docx]

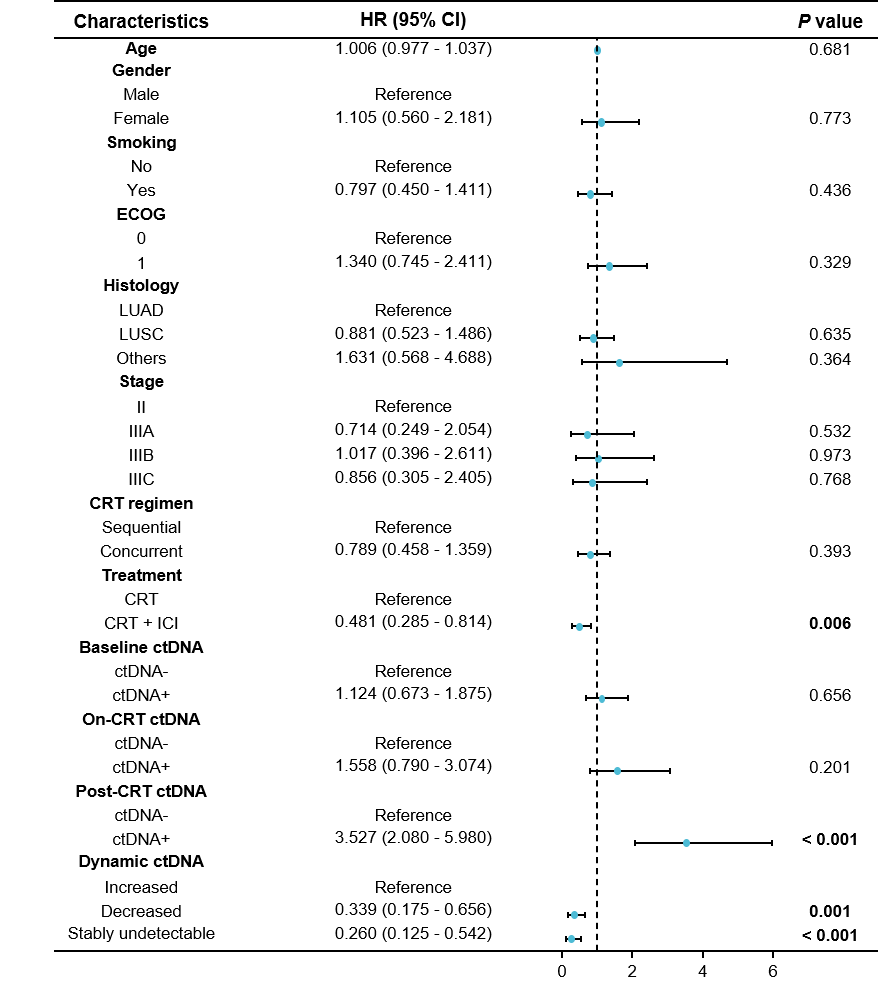


Figure S1. Forest plots of univariate cox proportional hazards regression analysis for predicting progression-free survival. HR, hazard ratio; CI, confidence interval; ECOG, Eastern Cooperative Oncology Group; CRT, chemoradiotherapy; ICI, immune checkpoint inhibitor; ctDNA, circulating tumor DNA.
