# Supplementary material for: Longitudinal circulating tumour DNA dynamics predict failure patterns and efficacy of consolidation immunotherapy after chemoradiotherapy in locally advanced non‐small‐cell lung cancer
Source: Clin Transl Med. 2024 Mar 7;14(3):e1619. doi: 10.1002/ctm2.1619 (PMC10918705; doi:10.1002/ctm2.1619)
Supplement: Supplementary file 2 — Supporting Information [file CTM2-14-e1619-s002.docx]

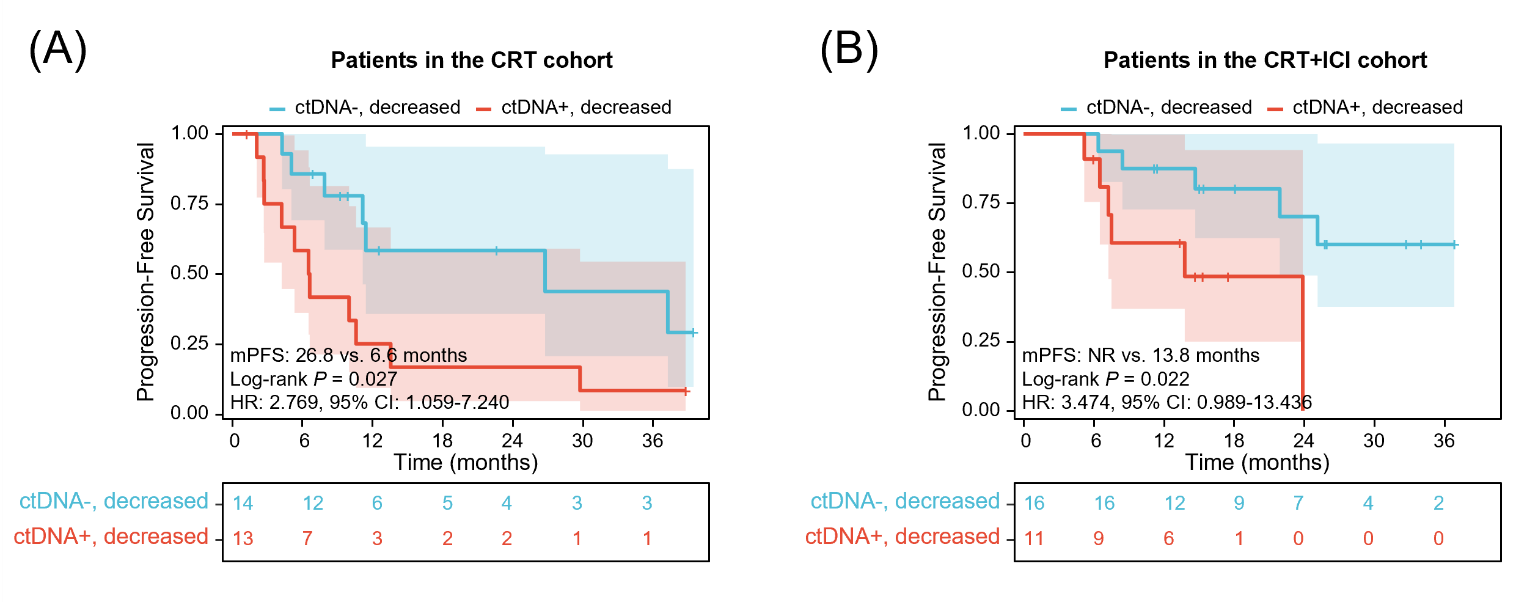


Figure S2. The predictive value of combing post-CRT ctDNA detection with dynamic ctDNA. (A) PFS stratified by patients with undetectable/decreased ctDNA (n=14) versus detectable/decreased ctDNA (n=13) in the CRT cohort. (B) PFS stratified by patients with undetectable/decreased ctDNA (n=16) versus detectable/decreased ctDNA (n=11) in the CRT and consolidation ICI cohort. mPFS, median progression-free survival; HR, hazard ratio; CI, confidence interval; NR, not reached.
