# Supplementary material for: Longitudinal circulating tumour DNA dynamics predict failure patterns and efficacy of consolidation immunotherapy after chemoradiotherapy in locally advanced non‐small‐cell lung cancer
Source: Clin Transl Med. 2024 Mar 7;14(3):e1619. doi: 10.1002/ctm2.1619 (PMC10918705; doi:10.1002/ctm2.1619)
Supplement: Supplementary file 3 — Supporting Information [file CTM2-14-e1619-s003.docx]

**Methods**

***Study design and patient population***

This prospective, multicenter cohort study was conducted in Cancer Hospital of Chinese Academy of Medical Sciences, Peking Union Medical College (Beijing) and Shenzhen Cancer Hospital (Shenzhen) in China from July 2018 to November 2022 (NCT04014465). Treatment-naïve patients with pathologically confirmed unresectable stage II or III non-small-cell lung cancer (NSCLC), according to the 8th edition of American Joint Committee on Cancer, were consecutively enrolled. Key exclusion criteria included patients with driver gene mutations, such as epidermal growth factor receptor or anaplastic lymphoma kinase. Eligible participants were assigned to the chemoradiotherapy (CRT) cohort (definitive concurrent or sequential CRT) or CRT + immune checkpoint inhibitor (ICI) cohort (CRT followed by consolidation ICI for up to 1 year or until progression).

Peripheral blood samples were collected from all patients at baseline, on-CRT (radiotherapy reached 40 Gy/20 fractions/4 weeks), post-CRT (1 month after the completion of CRT), and at the time of first disease progression, to profile longitudinal circulating tumor DNA (ctDNA) dynamics. Plasma specimens were analyzed with a next-generation sequencing panel covering 486 cancer-associated genes for ctDNA assessments.

***Treatment regimens and follow-up***

All patients underwent four-dimensional computed tomography (CT) simulation and were treated with intensity modulated radiation therapy or volumetric modulated arc therapy techniques with the prescribed dose of 56-66 Gy, concurrently or sequentially with ≥ 2 cycles of platinum-based doublet chemotherapy. Patients in the CRT + ICI cohort were further treated with consolidation programmed death 1 or programmed death ligand-1 (PD-L1) inhibitors after the completion of definitive CRT. Consolidation ICI treatment was administrated for up to 1 year or discontinued for progression or unacceptable toxicity.

All patients received pretreatment imaging examinations, including whole-body positron emission tomography (PET)-CT, or bone scintigraphy, brain magnetic resonance imaging, and CT of the neck, chest, and abdomen. Radiological follow-up was performed every 3 months for the first 2 years after CRT, every 6 months for the third to fifth year, and every year thereafter. Tumor response evaluations were assessed using CT or PET-CT, according to Response Evaluation Criteria in Solid Tumors (RECIST) version 1.1.

***DNA extraction and library construction***

The plasma fraction of peripheral blood samples (8-10 mL) was subjected to circulating free DNA (cfDNA) extraction with a Qiagen QIAamp Circulating Nucleic Acid Kit (Qiagen, Dusseldorf, Germany).^1^ Purified cfDNA samples were qualified using Nanodrop2000 (Thermo Fisher Scientific, Waltham, MA) and quantified using Qubit 2.0 dsDNA HS Assay Kit (Life Technologies, Waltham, MA). Libraries were prepared using the KAPA Hyper Prep Kit (KAPA Biosystems, Wilmington, MA).^1^ ∼50 ng of cfDNA was sequentially underwent end-repairing, A-tailing, and ligation with indexed adapters, followed by size selection and polymerase chain reaction (PCR) amplification with KAPA Hyper DNA Library Prep Kit (KAPA Biosystems). Target enrichment was performed using customized xGen lockdown probes (Integrated DNA Technologies) targeting 486 cancer-related genes (Radiotron, Nanjing Geneseeq Technology, China). Hybridization capture reaction was performed with Dynabeads M-279 (Life Technologies) and xGen Lockdown Hybridization and Wash Kit (Integrated DNA Technologies) per manufacturer’s instructions. Captured libraries were on-beads PCR amplified with Illumina p5 and p7 primers in KAPA HiFi HotStart ReadyMix (KAPA Biosystems), followed by purification using Agencourt AMPure XP beads. Libraries were quantified by quantitative PCR using KAPA Library Quantification kit (KAPA Biosystems). Library fragment size was determined by Bioanalyzer 2100 (Agilent Technologies).

***Next-generation sequencing and data processing***

Sequencing was performed on the Illumina HiSeq4000 platform. Sequencing data were analyzed by Trimmomatic to remove low-quality (quality < 15) or N bases,^2^ and then mapped to the human reference genome hg19 using the Burrows-Wheeler Aligner. PCR duplicates were removed by Picard. The Genome Analysis Toolkit (GATK) was used to perform local realignments around indels and base quality reassurance. Single nucleotide polymorphisms (SNPs) and indels were analyzed by VarScan2 and Haplotype Caller/Uni edGenotyper in GATK, with the mutant allele frequency cutoff of 0.2% for cfDNA samples, and a minimum of three unique mutant reads. Common SNPs were excluded if they were present in > 1% population frequency in the 1000 Genomes Project or the Exome Aggregation Consortium (ExAC) 65,000 exomes database. We applied CH-filtering and sequence white blood cell to manage the confounding factor of clonal hematopoiesis. The resulting mutation list was further filtered by an in-house list of recurrent artifacts based on a normal pool of whole blood samples. Gene fusions were identified by FACTERA.^3^ ctDNA levels were calculated based on previous studies: ctDNA abundance (ng/mL) = max ctDNA allele frequency × cfDNA concentration (ng/mL), ctDNA concentration (hGE/mL) = mean ctDNA allele frequency × cfDNA concentration (ng/mL) × 1000 ∕ 3.3.^4,5^

***Study endpoints and statistical analysis***

The primary endpoint was progression-free survival (PFS), defined as the time from diagnosis to the date of the first documented event of disease progression or death without progression, based on RECIST v1.1. The secondary endpoint was overall survival (OS), defined as the time from diagnosis until death from any cause, and distant metastasis-free survival (DMFS), defined as the time from diagnosis to first distant metastasis or death from disease. Survival time was calculated with the Kaplan-Meier method and compared using the log-rank test. Cox regression modeling was used to estimate hazard ratio (HR) and 95% confidence interval (CI). Proportions of categorical variables and distributions of continuous variables between groups were compared by Fisher’s exact tests and Mann-Whitney U-tests, respectively. Paired continuous data were compared using Wilcoxon signed-rank tests. Time-dependent receiver operating characteristic curves (ROCs) and areas under the curve (AUCs) were employed to assess the predictive power. Decision curve analysis (DCA) and concordance index (C-index) were performed to evaluate and compare the usefulness of different predictive models with respect to clinical decision-making benefit. Two-sided *P* value <0.05 was considered statistically significant. Statistics were analyzed using R software (version 3.5.1).

**References:**

1. Yang Z, Yang N, Ou Q, et al. Investigating Novel Resistance Mechanisms to Third-Generation EGFR Tyrosine Kinase Inhibitor Osimertinib in Non-Small Cell Lung Cancer Patients. *Clinical cancer research : an official journal of the American Association for Cancer Research*. Jul 1 2018;24(13):3097-3107. doi:10.1158/1078-0432.Ccr-17-2310

2. Bolger AM, Lohse M, Usadel B. Trimmomatic: a flexible trimmer for Illumina sequence data. *Bioinformatics (Oxford, England)*. Aug 1 2014;30(15):2114-20. doi:10.1093/bioinformatics/btu170

3. Newman AM, Bratman SV, Stehr H, et al. FACTERA: a practical method for the discovery of genomic rearrangements at breakpoint resolution. *Bioinformatics (Oxford, England)*. Dec 1 2014;30(23):3390-3. doi:10.1093/bioinformatics/btu549

4. Chaudhuri AA, Chabon JJ, Lovejoy AF, et al. Early Detection of Molecular Residual Disease in Localized Lung Cancer by Circulating Tumor DNA Profiling. *Cancer discovery*. Dec 2017;7(12):1394-1403. doi:10.1158/2159-8290.Cd-17-0716

5. Mao X, Zhang Z, Zheng X, et al. Capture-Based Targeted Ultradeep Sequencing in Paired Tissue and Plasma Samples Demonstrates Differential Subclonal ctDNA-Releasing Capability in Advanced Lung Cancer. *Journal of thoracic oncology : official publication of the International Association for the Study of Lung Cancer*. Apr 2017;12(4):663-672. doi:10.1016/j.jtho.2016.11.2235
