# Supplementary material for: Longitudinal circulating tumour DNA dynamics predict failure patterns and efficacy of consolidation immunotherapy after chemoradiotherapy in locally advanced non‐small‐cell lung cancer
Source: Clin Transl Med. 2024 Mar 7;14(3):e1619. doi: 10.1002/ctm2.1619 (PMC10918705; doi:10.1002/ctm2.1619)
Supplement: Supplementary file 4 — Supporting Information [file CTM2-14-e1619-s005.docx]

Table S1. Baseline characteristics of 105 included patients

| Characteristics | Overall | CRT | CRT + ICI | *P* value |
| --- | --- | --- | --- | --- |
|  | **N = 105** | **N = 55** | **N = 50** |  |
| **Age** (years), median (IQR) | 63 (55-67) | 64 (55-67) | 63 (58-67) | 0.921 |
| **Gender**, n (%)  Male  Female | 87 (83)  18 (17) | 46 (84)  9 (16) | 41 (82)  9 (18) | 0.824 |
| **Smoking**, n (%)  Current/former  Never | 78 (74)  27 (26) | 40 (73)  15 (27) | 38 (76)  12 (24) | 0.702 |
| **ECOG**, n (%)  0  1 | 33 (31)  72 (69) | 15 (27)  40 (73) | 18 (36)  32 (64) | 0.336 |
| **Histology**, n (%)  Squamous  Adenocarcinoma  Other | 56 (53)  44 (42)  5 (5) | 29 (53)  23 (42)  3 (5) | 27 (54)  21 (42)  2 (4) | 0.940 |
| **Stage**, n (%)  II  IIIA  IIIB  IIIC | 8 (8)  20 (19)  56 (53)  21 (20) | 6 (311)  9 (16)  29 (53)  11 (20) | 2 (4)  11 (22)  27 (54)  10 (20) | 0.555 |
| **CRT regimen**, n (%)  Concurrent CRT  Sequential CRT | 77 (73)  28 (27) | 36 (65)  19 (35) | 41 (82)  9 (18) | 0.056 |
| **Baseline ctDNA test**, median (IQR)  cfDNA abundance (ng/mL)  ctDNA abundance (ng/mL)  ctDNA concentration (he/mL)  Max VAF (%)  Mean VAF (%) | 17.6 (13.1-24.4)  0.2 (0-0.7)  41.1 (0-106.3)  1.0 (0-4.3)  0.8 (0-2.4) | 20.5 (14.6-25.8)  0.2 (0-0.7)  41.9 (0-111.8)  1.3 (0-4.1)  1.0 (0-2.6) | 14.8 (11.4-22.6)  0.2 (0-0.6)  40.2 (0-78.0)  0.9 (0-4.3)  0.7 (0-1.4) | 0.055  0.497  0.884  0.772  0.491 |

*Abbreviations*: CRT, chemoradiotherapy; ICI, immune checkpoint inhibitor; IQR, inter quartile range; ECOG, Eastern Cooperative Oncology Group; cfDNA, cell-free DNA; ctDNA, circulating tumor DNA; VAF, variant allele frequency. *P* values were derived based on the comparison of baseline characteristics between the CRT cohort and the CRT+ICI cohort.
