# Supplementary material for: Longitudinal circulating tumour DNA dynamics predict failure patterns and efficacy of consolidation immunotherapy after chemoradiotherapy in locally advanced non‐small‐cell lung cancer
Source: Clin Transl Med. 2024 Mar 7;14(3):e1619. doi: 10.1002/ctm2.1619 (PMC10918705; doi:10.1002/ctm2.1619)
Supplement: Supplementary file 5 — Supporting Information [file CTM2-14-e1619-s004.docx]

Table S2. Univariate cox regression models for progression-free survival

| Characteristics | Hazard ratio (95% CI) | *P* value |
| --- | --- | --- |
| **Age** | 1.006 (0.977-1.037) | 0.681 |
| **Gender**  Male  Female | Reference  1.105 (0.560-2.181) | 0.773 |
| **Smoking**  No  Yes | Reference  0.797 (0.450-1.411) | 0.436 |
| **ECOG**  0  1 | Reference  1.340 (0.745-2.411) | 0.329 |
| **Histology**  Adenocarcinoma  Squamous  Other | Reference  0.881 (0.523-1.486)  1.631 (0.568-4.688) | 0.635  0.364 |
| **Stage**  II  IIIA  IIIB  IIIC | Reference  0.714 (0.249-2.054)  1.017 (0.396-2.611)  0.856 (0.305-2.405) | 0.532  0.973  0.768 |
| **CRT regimen**  Sequential  Concurrent | Reference  0.789 (0.458-1.359) | 0.393 |
| **Treatment**  CRT  CRT+ICI | Reference  0.481 (0.285-0.814) | **0.006** |
| **Baseline ctDNA**  ctDNA-  ctDNA+ | Reference  1.124 (0.673-1.875) | 0.656 |
| **On-CRT ctDNA**  ctDNA-  ctDNA+ | Reference  1.558 (0.790-3.074) | 0.201 |
| **Post-CRT ctDNA**  ctDNA-  ctDNA+ | Reference  3.527 (2.080-5.980) | **< 0.001** |
| **Dynamic ctDNA post CRT**  Increased  Decreased  Stably undetectable | Reference  0.339 (0.175-0.656)  0.260 (0.125-0.542) | **0.001**  **< 0.001** |

*Abbreviations:* CI, confidence interval; ECOG, Eastern Cooperative Oncology Group; CRT, chemoradiotherapy; ICI, immune checkpoint inhibitor; ctDNA, circulating tumor DNA.
